# *Porphyromonas gingivalis* infection increases osteoclastic bone resorption and osteoblastic bone formation in a periodontitis mouse model

**DOI:** 10.1186/1472-6831-14-89

**Published:** 2014-07-15

**Authors:** Wenjian Zhang, Jun Ju, Todd Rigney, Gena Tribble

**Affiliations:** 1 Department of Diagnostic and Biomedical Sciences, 7500 Cambridge Street, Suite 5366, Houston 77054, TX, USA; 2 Department of Periodontics and Dental Hygiene, University of Texas School of Dentistry at Houston, 7500 Cambridge Street, Houston 77054, TX, USA

**Keywords:** Osteoblasts, *P. gingivalis*, Invasion, Micro-CT, Bone histomorphometry

## Abstract

**Background:**

*Porphyromonas gingivalis* has been shown to invade osteoblasts and inhibit their differentiation and mineralization in vitro. However, it is unclear if *P. gingivalis* can invade osteoblasts in vivo and how this would affect alveolar osteoblast/osteoclast dynamics. This study aims to answer these questions using a periodontitis mouse model under repetitive *P. gingivalis* inoculations.

**Methods:**

For 3-month-old BALB/cByJ female mice, 10^9^ CFU of *P. gingivalis* were inoculated onto the gingival margin of maxillary molars 4 times at 2-day intervals. After 2 weeks, another 4 inoculations at 2-day intervals were applied. Calcein was injected 7 and 2 days before sacrificing animals to label the newly formed bone. Four weeks after final inoculation, mice were sacrificed and maxilla collected. Immunohistochemistry, micro-CT, and bone histomorphometry were performed on the specimens. Sham infection with only vehicle was the control.

**Results:**

*P. gingivalis* was found to invade gingival epithelia, periodontal ligament fibroblasts, and alveolar osteoblasts. Micro-CT showed alveolar bone resorption and significant reduction of bone mineral density and content in the infected mice compared to the controls. Bone histomorphometry showed a decrease in osteoblasts, an increase in osteoclasts and bone resorption, and a surprisingly increased osteoblastic bone formation in the infected mice compared to the controls.

**Conclusions:**

*P. gingivalis* invades alveolar osteoblasts in the periodontitis mouse model and cause alveolar bone loss. Although *P. gingivalis* appears to suppress osteoblast pool and enhance osteoclastic bone resorption, the bone formation capacity is temporarily elevated in the infected mice, possibly via some anti-microbial compensational mechanisms.

## Background

Periodontitis affects up to 20% of the world’s population [1]. It is an indication of poor oral health and is linked to poor general health and a compromised quality of life, especially in geriatric population [2] . The hallmark of periodontitis is loss of connectivity between the tooth, periodontal tissues, and alveolar bone. Destruction of tissue and resorption of bone results in formation of the periodontal pocket, an enlarged subgingival space that is a protective habitat for periodontal microorganisms. The development of periodontitis is a multifactorial process revolving around complex host-microorganism interactions [3]. *P. gingivalis* is a gram-negative black pigmented anaerobe that colonizes the subgingival crevice, and has been identified as one of the major periodontal pathogens. It has multiple known virulence factors that contribute to its survival in the oral environment, such as fimbriae, gingipains, lipopolysaccharides (LPS), capsule, and hemagglutinins [4].

Ample evidence indicates that multiple components of *P. gingivalis* can act upon osteoblasts to inhibit alveolar bone formation. *P. gingivalis* LPS, lipids, metabolic products and sonicated extracts can inhibit the differentiation and osteogenesis of osteoblasts [5-10], and modulate RANKL (receptor activator of nuclear factor-kappaB ligand) and/or OPG (osteoprotegerin) expression in osteoblasts to indirectly stimulate osteoclastogenesis [11-14]. Recently, our laboratory has established that *P. gingivalis* can invade osteoblasts and inhibit their maturation and mineralization in vitro [15], and fimbriae play an important role in mediating the initial invasive process [16].

Periodontal pathogens have been shown to intrude upon alveolar surface and occupy empty lacunae in patients with severe periodontitis [17]. It is not clear if *P. gingivalis* can invade osteoblasts in vivo, and if so, how this would influence bone homeostasis at infected sites. Mice do not have *P. gingivalis* as part of their endogenous oral microflora [18]. However, periodontitis and alveolar bone loss have been induced successfully in mice by intraoral inoculation of live *P. gingivalis*[19-21]. The goal of the present study is to investigate the invasion of alveolar osteoblasts by *P. gingivalis*, and how osteoblast-osteoclast coupling is affected by bacterial infection in a periodontitis mouse model under repetitive *P. gingivalis* inoculations. It was found that *P. gingivalis* was able to invade periodontal cells and disturb the homeostasis of osteoblasts and osteoclasts, which ultimately contributes to alveolar bone loss.

## Methods

### Bacteria and culture conditions

*P. gingivalis* strain ATCC 33277 was grown anaerobically at 37°C in a Coy anaerobic chamber under an atmosphere of 86% nitrogen: 10% carbon dioxide: 4% hydrogen. Culture media was Trypticase Soy Broth (TSBY) supplemented with 5% yeast extract, 2% sodium bicarbonate, 7.5 μM hemin and 3 μM menadione. TSB blood agar plates (BAP) were made with the addition of 5% sheep’s blood and 1.5% agar. The bacteria were inoculated from BAP into 5 ml of TSBY and cultured anaerobically for 18 to 24 hrs at 37°C, then diluted in TSBY and grown to early log phase. Bacterial cells were harvested by low-speed centrifugation, washed, and resuspended in phosphate buffered saline (PBS). The concentration of bacteria was determined with a spectrophotometer at an optical density of 600 nm (OD 1 = 10^9^*P. gingivalis* per ml). 10^9^ CFU of live *P. gingivalis* was collected and pelleted by low-speed centrifugation, then resuspended in 20 μl of PBS with 2% carboxymethylcellulose. The bacterial suspension was applied to the gingival margin of mouse maxillary molars as detailed below.

### Bacterial inoculations

A mouse periodontitis model was established with slight modification of the method described previously [22]. Briefly, 10–12 week-old BALB/cByJ female mice were kept in specific-pathogen free environment. They received kanamycin at 1 mg/ml in deionized water ad libitum for 7 days. Three days after the antibiotic treatment, the mice were put under brief isoflurane anaesthesia, and 10^9^ CFU of live *P. gingivalis* in 20 μl of PBS with 2% carboxymethylcellulose was applied to the gingival margin of mouse maxillary molars four times, 2 days apart. The mice were restrained from food and water intake for 1 hr after inoculation. We used an inoculation concentration of 1×10^9^ CFU, since our preliminary study showed that this concentration was sufficient to induce *P. gingivalis* invasion of periodontium and alveolar bone loss. Sham infected group received 20 μl of PBS with 2% carboxymethylcellulose alone. Two weeks later, another four doses (2 days apart) were applied to the mice. Four-week after the second oral challenge, the mice were sacrificed, and the maxillary specimens were collected for micro-CT and bone histomorphometry studies. For *P. gingivalis* invasion study, immunohistochemistry was performed on maxilla specimens collected 3 days after three times of bacterial inoculations. Ten animals each were included for both infected and sham groups for the aforementioned studies. All animal-related experiments were approved by the Center for Laboratory Animal Medicine and Care at the University of Texas Health Science Center at Houston (approved animal protocol HSC-AWC-10-145).

### Immunohistochemistry

Mice maxillary specimens were isolated, fixed in 10% neutral buffered formalin, decalcified in 3.4% sodium formate/15% formic acid, and embedded in paraffin. Antigen retrieval was performed using 4 N HCl for 10 min at 37°C. Endogenous peroxidase activity was blocked by incubating 10 min with 3% H_2_O_2_. Nonspecific proteins were blocked with DAKO protein block (Dako, Carpinteria, CA) for 30 min at room temperature (RT). Sections were incubated at RT for 1 h with 1:4000 rabbit anti- *P. gingivalis* polyclonal antibody. Secondary antibody and substrate staining were performed with DAKO LSAB + kit and liquid DAB + substrate-chromogen system (Dako). The slides were counterstained with hematoxylin. The number of osteoblasts with bacterial invasion was counted manually. For both *P. gingivalis*-infected and control groups, 10 samples were analyzed. The total number of osteoblasts lining alveolar bone surface on the section was counted. The formula to cacluate percentage of positive staining was: (number of brown-stained osteoblasts/total number of osteoblasts counted) ×100.

### Micro-CT

**Figure 1:**
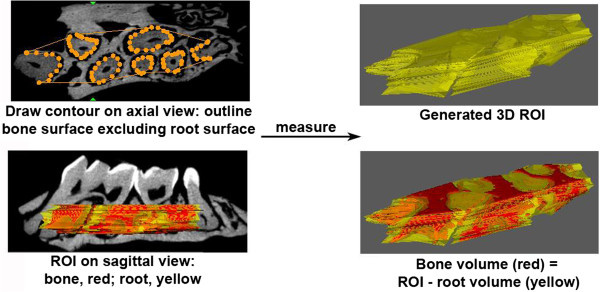
**Methods for generating 3D ROI (region of interest) used in micro-CT analysis of alveolar bone.** Two-dimensional contours were drawn manually every five data planes on the axial view, from the roof of the furcation to the root apex. Root surface area was excluded from the bone surface area on every measured plane. A 3D ROI was created after all the 2D contours were drawn. Alveolar bone volume was calculated as total ROI minus root volume.

Four weeks after the final inoculation, mouse maxilla were collected and imaged with a Scanco micro-CT 40 (SCANCO Medical, Brüttisellen, Switzerland) at a resolution of 12 micron. The micro-CT images were acquired at Baylor College of Medicine micro-CT Core Facility and reconstructed using a modified Feldkamp method [23]. The image data were analyzed similar to what was described by Park et al. [24]. Briefly, all images were reoriented such that the cement-enamel junction (CEJ) and root apex (RA) appear in the same slice. The region of interest (ROI) was drawn manually on the axial planes, between the medial root surface of the first molar and the distal root surface of the third molar. The contours were drawn continuously every 5 data planes from roof of the furcation all the way to the root apex, until a three-dimensional (3D) ROI is generated (Figure 1). All root volumes were excluded from the ROI to calculate the total volume (TV). The parameters analyzed for each sample included bone volume fraction (BVF = BV/TV), bone mineral density (BMD, normalized to a hydroxyl apatite phantom), and bone mineral content (BMC = BMDX BV).

### Bone histomorphometry

Four weeks after the final inoculation, mouse maxilla were collected and cut into two halves for static and dynamic histomorphometry analysis, respectively. For dynamic histomorphometry, mice received 10 mg/kg body weight of calcein in 2% NaHCO3 intraperitoneally 7 and 2 days before sacrificing to label the newly formed bone. Maxilla were dissected free of soft tissues and crowns of teeth were amputated along alveolar bone surface. Specimens were fixed in 70% ethanol for 3–5 days, dehydrated in increasing concentrations of ethanol, cleared in xylene, and embedded undecalcified in methyl methacrylate. Five micrometer thick sections were cut parallel with the long axis of the molars, and sections near the center of pulp chambers were mounted unstained for visualization of mineralizing surface under UV light with an I3 filter. For static measurement, maxilla was fixed in 4% paraformaldehyde in PBS, pH 7.4, at 4°C for 2–5 days. The bones were decalcified in EDTA/NH_3_OH for an additional 2–5 days and dehydrated in increasing concentrations of ethanol, cleared in xylene, and embedded in paraffin. The embedded bones were cut parallel with the long axis of the molars, and sections near the center of pulp chambers were mounted. Consecutive sections were stained with Toluidine blue (Sigma-Aldrich, St. Louis, MO) or Tartrate-resistant acid phosphatase (TRAP, Sigma-Aldrich) for visualization of osteoblasts and osteoclasts, respectively. The cell types were further validated by their characteristic morphology, such as cuboidal for osteoblasts and multinucleated for osteoclasts. All of the osteoblasts and osteoclasts on the entire section were counted, to avoid the spatial variations in their distributions. Histomorphometric measurements were made in a blinded, nonbiased manner, and the terminology and units used were those recommended by the Histomorphometry Nomenclature Committee of the American Society for Bone and Mineral Research [25]. All bone histomorphometry analysis was performed at the University of Texas MD Anderson Cancer Center Bone Histomorphometry Core Facility using the Bioquant Osteo II computerized image analysis system (BIOQUANT Image Analysis Corporation, Nashville, TN) interfaced with a Leica DM 1000 microscope (Leica Microsystems, Wetzlar, Germany).

### Statistics

*P. gingivalis*-infected and sham groups have 10 animals each for all the experiments. Statistical analysis was performed by Student’s t test to determine significance between groups (p < 0.05).

## Results

### *P. gingivalis* invades gingival epithelial cells, periodontal ligament (PDL) cells, and alveolar osteoblasts in the periodontitis mouse model

**Figure 2:**
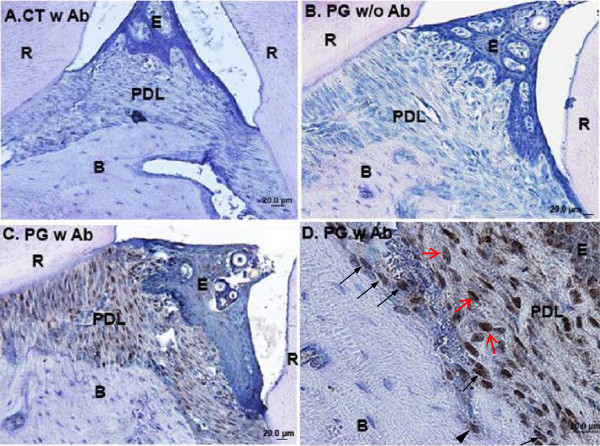
***P. gingivalis *invade periodontal soft and hard tissues after repetitive inoculations as shown by immunohistochemistry.** Immunohistochemistry was performed three days after three times of *P. gingivalis* inoculations. **A**. Sham-infected control. Anti-*P. gingivalis* primary antibody was applied in the assay. Blue is hematoxylin counterstaining. Notice there is no brown positive staining for *P. gingivalis* in the periodontium. **B**. *P. gingivalis* infected animals, with anti-*P. gingivalis* primary antibody excluded from the assay, which shows no positive staining in the periodontium. This demonstrated there was no unspecific staining from secondary antibody and/or substrate. **C**. *P. gingivalis* infected animals, with anti-*P. gingivalis* primary antibody included in the assay. Extensive staining for *P. gingivalis* was noticed in gingival epithelial cells, PDL fibroblasts, and alveolar osteoblasts. **D**. Magnified view in **C**. Positive *P. gingivalis* staining was detected in fibroblasts (denoted by red arrows) in periodontal ligament space, osteoblasts lining the alveolar bone surface (denoted by black arrows), and in an osteocyte embedded in the alveolar bone matrix (denoted by black arrow head). Abbreviations: CT, control, sham-infected; PG, *P. gingivalis* infected; Ab, antibody; R, root; E, gingival epithelial cells; PDL, periodontal ligament; B, alveolar bone; scale bar = 20 μm.

Three days after three times of oral challenge with *P. gingivalis*, immunohistochemistry showed positive staining for *P. gingivalis* in gingival epithelial cells, PDL fibroblasts, osteoblasts lining the alveolar surface, and osteocytes embedded in bone matrix in the infected group, but no staining was detected in the control sham-infected group (Figure 2). Approximately 17.6 ± 2.1% of alveolar osteoblasts had *P. gingivalis* invasion based on manual counting. Positively-stained (brown) periodontal fibroblasts were evenly distributed within the connective tissue. This result demonstrates that *P. gingivalis* successfully invades deep periodontium in our periodontitis animal model.

### *P. gingivalis* infection causes loss of alveolar bone volume and density

**Figure 3:**
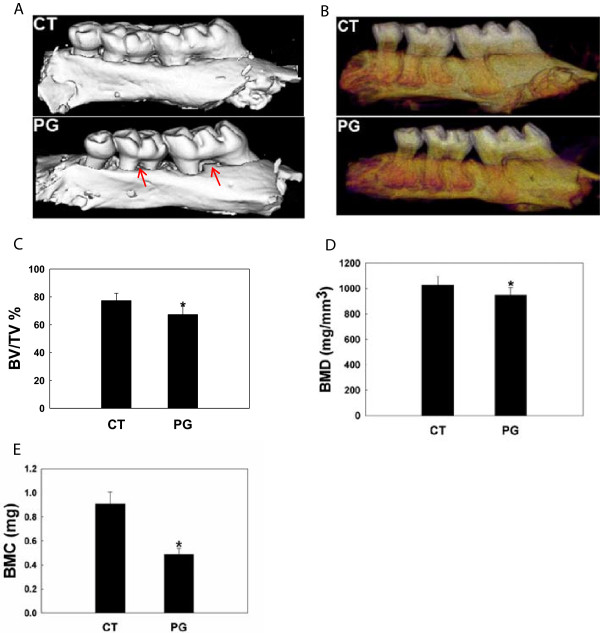
***P. gingivalis *infection causes loss of alveolar bone volume and density as demonstrated by micro-CT.** Micro-CT analysis was done four weeks after a total of eight bacterial inoculations. Decreased alveolar bone height and furcation involvement **(A)** and decreased mineral density at the alveolar surface **(B)** was noticed in the infected animals. In panel **A**, red arrows point to the furcation involvement. In panel **B**, the darker reddish-purple color indicates regions of lower mineral density. Quantification of micro-CT data shows that repetitive *P. gingivalis* infection caused a significant reduction in alveolar bone fraction **(C)**, bone mineral density **(D)**, and bone mineral content **(E)** compared with the controls. Abbreviations: CT, control, sham-infected; PG, *P. gingivalis* infected; BV/TV%, remaining alveolar bone volume over total volume; BMD, bone mineral density (normalized to hydroxyl apatite phantom); BMC, bone mineral content (=BMD X BV); *, denotes P < 0.05 compared with the controls.

Four weeks after the final oral inoculation with *P. gingivalis*, mouse maxilla specimens were collected and imaged with Micro-CT scanner. The Micro-CT images show decreased alveolar bone height and furcation resorption of mouse molars (Figure 3A), and decreased mineral density of alveolar bone especially at the gingival margin in the infected mice compared to the sham control (Figure 3B). Quantification of micro-CT results demonstrates significant reduction of alveolar bone volume fraction, bone mineral density, and bone mineral content of infected animals compared to the controls (Figure 3C-E).

### *P. gingivalis* infection results in an increase in osteoclastic bone resorption and a compensational increase in osteoblastic bone formation

**Figure 4:**
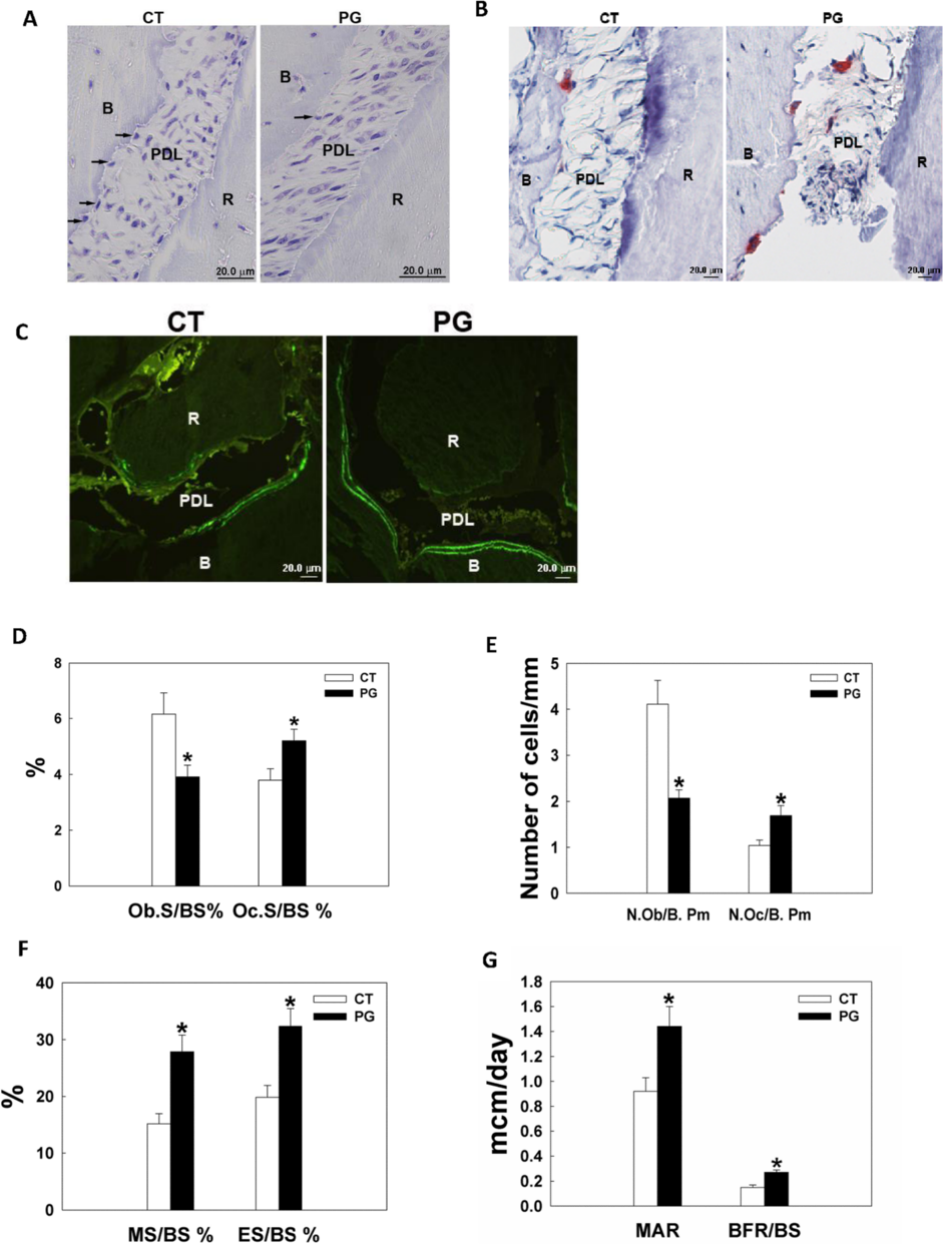
***P. gingivalis *infection results in increased osteoclastic bone resorption and increased osteoblastic bone formation as shown by bone histomorphometric analysis.** Bone histomorphometry was done four weeks after a total of eight bacterial inoculations. **A**. Toluidine blue staining for osteoblasts. Arrows denote osteoblasts in contact with the alveolar bone surface. **B**. TRAP staining for osteoclasts, which are red-stained multinucleated cells. **C**. Calcein doubling labeling to show newly formed bone. The area between the double bright green lines is where the new bone is formed. **D-G**, quantified bone histomorphometry data. A significant decrease of osteoblast number **(D and E)**, increased osteoclast number **(D and E)**, and increased osteoclastic bone resorption **(F)** was observed in the infected animals. Surprisingly, osteoblastic bone formation was greatly elevated in the remaining osteoblasts **(F and G)**. Abbreviations: CT, control, sham-infected; PG, *P. gingivalis* infected; B, alveolar bone; R, root; PDL, periodontal ligament; Ob.S/BS%, percent bone surface lined with osteoblasts; Oc.S/BS%, percent bone surface covered with osteoclasts; N.Ob/B. Pm, number of osteoblasts per bone perimeter; N. Oc/B. Pm, number of osteoclasts per bone perimeter; MS/BS%, percent of mineralizing surface of total bone surface measured; ES/BS%, percent bone surface eroded by osteoclasts; MAR, mineral apposition rate; BFR/BS, bone formation rate; *, denotes P < 0.05 compared with the controls. Scale bar = 20 μm.

To evaluate how osteoblast/osteoclast coupling was affected by *P. gingivalis*, bone histomorphometry analysis was carried out on the maxilla specimens. Bacterial infection caused a decrease in osteoblast number (Figure 4A, D, and E), an increase in osteoclast number (Figure 4B, D, and E) and elevated alveolar bone resorption (Figure 4F). Unexpected, osteoblastic bone formation was stimulated by *P. gingivalis*, as demonstrated by significantly higher MS/BS% (percent of mineralizing surface of total bone surface measured, Figure 4C and F), MAR (mineral apposition rate, Figure 4C and G), and BFR/BS (bone formation rate, Figure 4C and G) in the infected group compared with the controls.

## Discussion

In this study, we investigated invasion of the periodontium by *P. gingivalis* and how bacterial infection influences alveolar bone dynamics using a periodontitis mouse model. Murine models have been used extensively to explore the pathogenesis of periodontal diseases and develop treatment modalities, because the periodontal anatomy and histopathology of periodontal lesions in mice are similar to those found in humans [26]. In addition, low cost, ease of handling, known genetics, a well-defined immune system, and controllable oral microflora favor murine over other animal species in periodontal studies [27,28]. Different methods have been presented in the literature for delivering periodontal pathogens to the oral cavity of experimental animals, such as with diet, bacteria-soaked ligature, gavage, or direct application of bacterial suspension to gingiva [18,21,26,29,30]. To achieve a site-specific infection and avoid gingival damage from ligature, we suspended live *P. gingivalis* in slightly viscous solution (2% carboxymethylcellulose in PBS) and applied it directly on the gingival margin of mouse maxillary molars.

Our periodontitis mouse model is a very simplified simulation of clinical periodontitis seen in the patient population. We used single *P. gingivalis* infection mainly to see if they can invade periodontium in vivo and cause periodontal bone loss. Since human gingival sulcus is a habitant for highly diversified oral microflora [31], further studies with polymicrobial periodontitis animal models would provide additional insight on the pathogenic mechanism of periodontal disease.

In vitro studies have shown that *P. gingivalis* can infiltrate human transformed and primary gingival epithelial cells as well as multilayered pocket epithelial cells [32-35]. Using an engineered human oral mucosa model composed of normal human epithelial cells and fibroblasts and a reconstituted basement membrane model (Matrigel), *P. gingivalis* has been shown to infiltrate multilayered epithelial cells, migrate through the basement membrane, and reach the underlying connective tissue [36]. In vivo studies have demonstrated the invasion of human crevicular and buccal epithelial cells [37-39], and tissue invasion of gingival biopsy specimens by *P. gingivalis*[40,41]. These results suggest that periodontal pathogens may penetrate deep periodontal tissues in vivo. Our immunohistochemistry study shows invasion of gingival epithelial cells, fibroblasts, alveolar bone lining osteoblasts, and embedded osteocytes by *P. gingivalis* at the infected sites 3 days after multiple oral inoculations. This is the first study to demonstrate that this periodontal pathogen is capable of invading alveolar osteoblasts in vivo. Multiple virulence factors of *P. gingivalis*, especially fimbriae and gingipains, may play an important role in facilitating intracellular invasion and extracellular breakdown and, ultimately, progression and expansion of periodontal damages [4,42,43]. Our previous in vitro study demonstrates that fimbriae are essential for initial invasion of *P. gingivalis* into osteoblasts, but may not be so critical for the persistence of bacteria inside host cells [16].

To demonstrate initial colonization/infection of *P. gingivalis* in mouse oral cavity, a short-term immunohistochemistry study was performed to evaluate the invasion of periodontal cells by *P. gingivalis* three days after three times of bacterial inoculations. This early phase was chosen because we wanted to demonstrate that the presence of *P. gingivalis* inside of periodontal cells was mainly due to invasion instead of intracellular multiplication of the bacteria. Immunohistochemical staining demonstrated entry of *P. gingivalis* into different periodontal cells. However, within this short time window, no appreciable apical migration of junctional epithelium and pocket formation was noticed. Apparent alveolar bone loss was not detected until much later, which was 4 weeks after a total of 8 inoculations. No obvious histological signs of inflammation, such as infiltration of inflammatory (polymorphonuclear leukocytes, lymphocytes) cells in the connective tissue, was demonstrated in our early phase immunohistochemistry study. This probably was due to the intrinsic low ability of *P. gingivalis* to stimulate host immune/inflammatory responses [44-47]. Cell wall extracts and purified cellular components from *P. gingivalis* have comparatively weak host immunostimulatory activity [44-47]. *P. gingivalis* can actively inhibit the secretion of neutrophil chemoattractant Interleukin (IL)-8, IL-1β, and IL-18 [48,49]. Perhaps, the suppression of host innate immune & inflammatory responses by *P. gingivalis* could facilitate the maintenance of the chronic state of infection during periodontal diseases. Bacteria other than *P. gingivalis* in the polymicrobial oral flora seem to be able to mediate very robust host immune & inflammatory responses [30,49]. Our results demonstrated that direct invasion of *P. gingivalis* into osteoblasts suppressed osteoblast pool and disrupted osteoblast/osteoclast hemostasis resulting in a net bone loss. However, alveolar bone destruction due to host inflammatory reaction to *P. gingivalis* cannot be ruled out.

*P. gingivalis* invasion of periodontal cells was demonstrated as specific intracellular brown staining for the bacteria, which was absent in control groups. Toluidine blue staining on consecutive tissue sections confirms that the cuboidal-shaped cells on the alveolar bone surface were truly osteoblasts (data not shown). With the power of light microscope that we used to capture the images, individual bacterium cannot be resolved. Instead, the cells with bacterial invasion demonstrated punctuate or diffuse brown cytoplasmic staining. Future transmission electron microscopy study may further pinpoint the subcellular localization of the bacteria.

Our micro-CT study demonstrates significantly decreased residual alveolar bone volume and mineral density in the *P. gingivalis*-infected animals compared with the sham-infected controls, which is consistent with previous reports [30,50]. The alveolar bone density appeared to decrease more at the gingival margin than rest of the area in the infected animals (Figure 3B), probably due to close proximity to the bacteria and their secreted virulence factors at these surfaces.

The bone histomorphometry analysis revealed elevated osteoclast number and osteoclastic bone resorption in the infected animals compared to the controls, which is similar to other findings [50]. Interestingly, for osteoblast parameters, significantly decreased osteoblast numbers but significantly increased bone formation activity was noticed in the infected animals. Our previous in vitro study demonstrates that *P. gingivalis* initially suppresses but later promotes osteoblast apoptosis upon repetitive inoculations [51] . Considering the time course of the in vivo study, the decreased osteoblast number could be due to increased apoptosis and/or inhibition of osteoblastogenesis by the bacteria. At the examined time point, although the total number of osteoblasts was reduced, it appeared that a larger fraction of residual osteoblasts was stimulated to actively lay down bone compared with controls, possible due to some compensational mechanisms in response to the bacterial challenge. However, the increase in bone formation was outweighed by the increase in bone resorption, therefore, a net bone loss was observed in the infected animals. It appears that the reaction of alveolar bone to this periodontal pathogen is complicated, and further longitudinal bone histomorphometric analysis would be helpful to better unravel the disease’s mechanism.

In terms of what are the actual intracellular effects of *P. gingivalis* in osteoblasts/osteoclasts, our previous in vitro study demonstrates that invasion of *P. gingivalis* does not affect osteoblast proliferation, but inhibits their differentiation and mineralization, partially via an inhibition of the differentiation regulatory transcription factors Cbfa-1 and osterix [15]. Another in vitro study finds that binding between *P. gingivalis* fimbriae and osteoblast integrins alph5beta1 results in periphery condensation of actin and activation of JNK pathway in the infected osteoblasts [51]. Our preliminary osteoblast-osteoclast coculture study demonstrates increased RANKL and decreased OPG concentrations in *P. gingivalis* infected cocultures compared with the control, resulting in a much higher RANKL/OPG ration which may stimulate osteoclastogenesis and subsequent bone resorption (unpublished data). Apparently, more thorough studies are needed to identify additional host-pathogen interactive molecules (adhesins, cytokines, et al.) in periodontitis, to facilitate the development of new therapeutic targets to prevent periodontal bone loss and/or to stimulate regeneration of alveolar bone.

## Conclusions

In summary, the data presented herein demonstrates that *P. gingivalis* can invade alveolar osteoblasts, cause a decrease in osteoblasts, an increase in osteoclasts and bone resorption, and an unexpected increase in bone formation in the infected mice compared to the controls. The bone resorption outweighs new bone formation therefore the infected mice demonstrate an overall reduced alveolar bone volume and density. Having a better understanding of the pathogenic mechanisms of periodontitis may lead to the development of new preventive or therapeutic strategies against this refractory disease.

## Abbreviations

*P. gingivalis*: *Porphyromonas gingivalis*; CFU: Colony-forming unit; LPS: Lipopolysaccharides; RANKL: Receptor activator of nuclear factor-kappaB ligand; OPG: Osteoprotegerin; TSBY: Trypticase Soy Broth supplemented with yeast extract; BAP: Blood agar plates; PBS: Phosphate buffered saline; OD: Optical density; RT: Room temperature; CEJ: Cement-enamel junction; RA: Root apex; ROI: Region of interest; 3-D: Three-dimensional; BV: Bone volume; BVF: Bone volume fraction; BMD: Bone mineral density; BMC: Bone mineral content; TRAP: Tartrate-resistant acid phosphatase; PDL: Periodontal ligament; 2D: Two-dimensional; MS/BS%: Percent of mineralizing surface of total bone surface measured; MAR: Mineral apposition rate; BFR/BS: Bone formation rate; IL: Interleukin; CT: Control, sham-infected; PG: *P. gingivalis* infected; Ab: Antibody; R: Root; E: Gingival epithelial cells; B: Alveolar bone; Ob.S/BS%: Percent bone surface lined with osteoblasts; Oc.S/BS%: Percent bone surface covered with osteoclasts; N.Ob/B. Pm: Number of osteoblasts per bone perimeter; N. Oc/B. Pm: Number of osteoclasts per bone perimeter; ES/BS%: Percent bone surface eroded by osteoclasts.

## Competing interests

The authors declare that they have no competing interests.

## Authors’ contributions

WZ conceived of the study, participated in its design and data interpretation, and drafted the manuscript. JJ carried out animal studies and specimen collection and preparation. TR carried out microbiology studies. GT helped with study design and data interpretation, and critically revised the manuscript. All authors read and approved the final manuscript.

## Pre-publication history

The pre-publication history for this paper can be accessed here:

http://www.biomedcentral.com/1472-6831/14/89/prepub
